# Maternal health development programs: comparing priorities of bilateral and private donors

**DOI:** 10.1186/s12914-014-0031-x

**Published:** 2014-11-19

**Authors:** Cécile Deleye, Achim Lang

**Affiliations:** SEEK Development, Greifswalder Str. 33A, 10405 Berlin, Germany; Department of Politics and Public Administration, University of Konstanz, Postbox 5560 D81, 78457 Konstanz, Germany

**Keywords:** Maternal health, Donors, Development projects, Corporations, Foundations

## Abstract

**Background:**

The face of international aid for health and development is changing. Private donors such as foundations and corporations are playing an increasingly important role, working in international development as direct operators or in partnerships with governments. This study compares maternal health programs of new development actors to traditional governmental donors. It aims to investigate what maternal health programs large governmental donors, foundations and corporate donors are conducting, and how and why they differ.

**Methods:**

A total of 263 projects were identified and analyzed. We focus on nine categories of maternal health programs: family planning services, focus on specific diseases, focus on capacity building, use of information and communication technology (ICT), support of research initiatives, cooperation with local non-state or state partners and cooperation with non-local non-state or state partners. Data analysis was carried out using Generalized Linear Mixed-Effects Models (GLMER).

**Results:**

Maternal health policies of public and private donors differ with regard to strategic approaches, as can be seen in their diverging positions regarding disease focus, family planning services, capacity building, and partner choice. Bilateral donors can be characterized as focusing on family planning services, specific diseases and capacity-building while disregarding research and ICT. Bilateral donors cooperate with local public authorities and with governments and NGOs from other developed countries. In contrast, corporations focus their donor activities on specific diseases, capacity-building and ICT while disregarding family planning services and research. Corporations cooperate with local and in particular with non-local non-state actors. Foundations can be characterized as focusing on family planning services and research, while disregarding specific diseases, capacity-building and ICT. Foundations cooperate less than other donors; but when they do, they cooperate in particular with non-state actors, local as well as non-local.

**Conclusions:**

These findings should help developing coordination mechanisms that embrace the differences and similarities of the different types of donors. As donor groups specialize in different contexts, NGOs and governments working on development and health aid may target donors groups that have specialized in certain issues.

## Background

Foreign aid from private sources is changing the landscape of international development assistance, adding to the work of traditional donors such as bilateral and multilateral organizations. Private donors are playing an increasingly important role, working on international development issues as direct operators, in partnership with governments and with international NGOs as grant providers. At the beginning of the twenty-first century, there are more philanthropic and corporate foundations holding more assets in more countries than ever before [1].

Funding priorities have increasingly come under scrutiny from the scientific community [2-5]. However, looking at funding allocations alone is not sufficient to assess the role of private actors in development. In fact, it is essential to get an understanding of their basic operating models and policies at the project level [6]. It is being assumed that organizations choose their projects according to their overall strategies and goals. Moreover, by looking at projects and not just formulated goals and strategies, the paper analyzes what types of maternal health projects are actually being conducted by the donors. This often provides a more accurate picture about the strategic priorities than official mission statements.

In the complex new donor landscape, mechanisms for coordination, information sharing and planning are crucial for effective development aid [7,8]. Knowing about differences and understanding them are critical preconditions for effective cooperation, collaboration and partnership, as it can be expected that the new actors come with different expertise and approaches. However, such a comparison of private and bilateral donors has not been conducted yet.

This study seeks to fill this research gap by analyzing the health programs of large bilateral donors, foundations and corporate donors in the area of maternal health, which can be defined as the health of “mothers and babies during pregnancy, childbirth and the postpartum period” [9]. Maternal health has become a focus area of the World Health Organization (WHO), as the death of a mother has long-term destabilizing effects on family and community structures in most developing countries [10]. Other focus areas of the WHO, such as HIV/AIDS or other infectious diseases, have already been studied for decades and clear funding and project preferences of public and private donors have been carved out. This is not the case for more long-term and less visible health issues such as maternal health. Thus, our analysis of maternal health projects broadens our understanding of donor preferences. Additionally, international and bilateral norm promotion and resource transfer are important drivers of domestic maternal health prioritization in developing countries, as Shiffman points out for several countries [11]. Moreover, the levels of maternal mortality are regarded as sensitive indicators of the entire health system, since interventions in these areas require a functioning health system to have an effect at the population level [12].

In this study, we delineate maternal health programs along nine categories: family planning services, focus on specific diseases, focus on capacity building, use of information and communication technology (ICT), support of research initiatives, cooperation with local non-state or state partners and cooperation with non-local non-state or state partners. The objects of analysis constitute 263 maternal health projects by fifteen donors that were conducted within the time frame between June 2012 and June 2013. The fifteen donors include the most resourceful five donors per category: bilateral/government, philanthropic foundations and corporate donors. The geographic focus of the projects was restricted to middle and low-income countries in Africa, Latin America, Oceania and Asia.

## Methods

The reason for using five bilateral donors, five foundations, and five corporate donors is that when choosing the largest donors from each group (measured by spending) and comparing their programs, it is possible to draw some conclusions on the likelihood that differences in policy design are due to donor characteristics. Obviously, the representativeness of the findings for smaller organizations is limited. However, it still might be possible to generalize. This assumption rests upon the fact that large organizations exert influence on the direction of aid and the priorities of organizations in general.

In our analysis, projects are the response variable and donor type is the predictor variable. Individual donor organizations enter the analysis as a grouping factor that represents unobserved organizational effects.

For bilateral donors, the OECD/DAC International Development Statistics (IDS) online database was used. We selected bilateral donors according to their commitments for total health and population policies/programs & reproductive health which include all maternal health interventions. This leads to the five largest bilateral donors: United States, United Kingdom, Canada, Australia and Germany.

Determining the largest foundations working in the area of maternal health is more challenging, since there is no global database with uniform spending data of foundations. The largest foundations organized by total amount of giving in 2011 were chosen according to total spending, because no sub data for maternal health programs were available. This sample selection procedure rests on the assumption that the largest donors also spend the most on maternal health projects. There might be smaller foundations, such as the MacArthur Foundation, that disproportionately spent on maternal health.^a^ However, in order to systematically select our sample we have to rely on comparable data. Two spending databases were taken into consideration: the Global Health Visions Landscape Analysis from 2011 and the US Foundation Center database from 2013. The five largest foundations were: the Bill and Melinda Gates Foundation, the Wellcome Trust, the Ford Foundation, the William and Flora Hewlett Foundation and the David and Lucile Packard Foundation. All of these foundations are grant making foundations. It was assumed that they are only funding projects that are aligned with their priorities. However, only grants for specific projects or pilot studies were included in the analysis, not grants for general support or organizational development of grantees, in order to enable a better comparison with bilateral donors.

Aid expenditures of corporations were analyzed using the Global Health Visions Landscape Analysis [13], research conducted by Christine Dugay [14], a report by Population Services International [15], and the members list of the Partnership for Maternal, Newborn and Child Health [16]. The five largest corporate donors were Johnson & Johnson, Merck and the Merck Company Foundation, the Abbott Fund, the Novartis Foundation for Sustainable Development and the Sanofi Espoir Foundation.

The first step in estimation of donor differences was to identify all relevant maternal health programs. The first criterion in the case selection procedure was to establish whether the goal of improving maternal health was included in the project name or description. Maternal health is inextricably linked to reproductive health and there are a number of definitions available for the two concepts [13]. The “Components of a Safe Motherhood Package” [17] served as a guideline for which specific interventions to include if maternal health was not stated as a goal in the project description, although it still appeared obvious that the project was a maternal health project. Programs regarding the prevention of mother-to-child transmission of HIV were not taken into account, since these mostly address the health of the child (although they have a positive effect on the mother’s health) and the issue of priorities in HIV/AIDS projects is a different topic. Newborn and child health were also often addressed in maternal health projects, but with these topics, only maternal health policies were analyzed. Furthermore, general health system strengthening projects were also not included, even though they indirectly affect the mother’s health, since only direct effects were of interest. Additionally, large product donation partnerships, awareness raising campaigns, coordination networks or alliances/coalitions such as the WHO-based Partnership for Maternal, Newborn and Child Health that act globally were not taken into account if they did not conduct tangible projects. Contributions by bilateral donors to multilateral organizations were not included for the same reason. The grant amount or project costs were also not of interest, as these constitute a different kind of analysis that doesn’t focus on actual policies. Public-Private Partnerships were also not a major focus of this analysis, as the differences between donor groups were of main interest instead. We furthermore restricted the geographic focus of projects to Latin America, Africa, Asia (except for Russia) and Oceania (except for Australia and New Zealand). Projects in North America and Europe were excluded; for example, grants from a foundation to an US grantee for promoting a maternal health campaign in the U.S., for organizational development of a European NGO or for organizing a conference on maternal health. We identified 263 maternal health projects that matched our criteria (See Table 1).

**Table 1:** **Number of projects per donor group and by donor**

**Bilateral**		**Foundations**		**Corporations**	
USA	41	Packard Foundation	26	Merck	24
UK	40	Ford Foundation	15	Johnson & Johnson	11
Canada	25	Gates Foundation	13	Sanofi Espoir Found	9
Australia	20	Hewlett Foundation	12	Novartis Foundation	4
Germany	15	Wellcome Trust	6	Abbott Fund	2
**Total**	**141**	**Total**	**72**	**Total**	**50**

Data on the maternal health projects was collected on the agencies’ or foundations’ websites. The project databases provide information such as project description, details about recipients and time periods. Moreover, spot-checks were made with the AidData website [18], a project run by several universities and non-profits to serve as a registry of aid activities to improve transparency.

We focus on nine categories of maternal health programs:

- **Family planning services** (FP) are defined as education, counseling and contraceptive commodities provided on a voluntary basis to females and couples [19,20]. FP services were coded with 1 if the services were actually offered, or if there was research, advocacy or consultation about it, as this means that the donor approves these programs. It may be that in some cases when maternal health services were listed in the project description, this included FP services. However, they were only coded with 1 if they were explicitly mentioned in the project description.
- **Disease-specific approaches** were coded with 1 if a concrete disease or medical condition was mentioned in the project description (e.g. fistula, postpartum hemorrhage, unsafe abortion, nutrition problems or malaria in pregnancy).
- **Capacity-building approach** is a process by which individuals, institutions and countries strengthen capacities or abilities, such as enhancing the skills, knowledge and social capabilities available to individuals, institutions, and social and political systems [21].
- **Use of ICT** means “the acquisition, analysis, manipulation, storage and distribution of information; and the design and provision of equipment and software for these purposes” [22]. ICT goods are intended to fulfill the function of information processing and communication by electronic means [23,24].
- A project was rated as providing **support for research initiatives** if it clearly stated in the project description that research was conducted, which is thought to be anything that involves scientific research methods, including pilot studies or tests of tools.
- **Cooperation with local partners (state)** and **cooperation with local partners (non-state):** A local partner is defined as being from and headquartered in the country where the project takes place in comparison to multilateral organizations or global NGOs that have country offices in many regions but still receive direction from their headquarters.
- **Cooperation with non-local non-state actors (NSAs) and governments:** This category includes donors’ work with other NSAs and governments that are not from the country where the project is located.

Data analysis was carried out using Generalized Linear Mixed-Effects Models (GLMER) available in the R programming environment within the packages *nlme* [25]. Such models provide an efficient means to model item level responses clustered within groups. As in every linear model, a GLMER describes the relationship between a response variable and certain covariates. In a mixed effects model, at least one of these covariates is categorical and represents a grouping factor. In this essay, the fifteen organizations were treated as a grouping factor. Random effects can be interpreted as representing unobserved random variables within the grouping factors such as unobserved organizational effects [25]. The GLMER estimates intercepts for each level of the grouping factor, in this case for every donor. Models also estimate the between donor variance which can be interpreted as the residual variability that is left over and that cannot be attributed to either the grouping factor or the fixed effects. In contrast, fixed effects represent the average (estimated) relationship between response and covariates. This provides the average or population model. In this analysis, we model the effects using the binomial family of the GLMER framework. Thus, we are interested in the probability that donor type has an effect on the design of a certain project.

## Results

**Figure 1:**
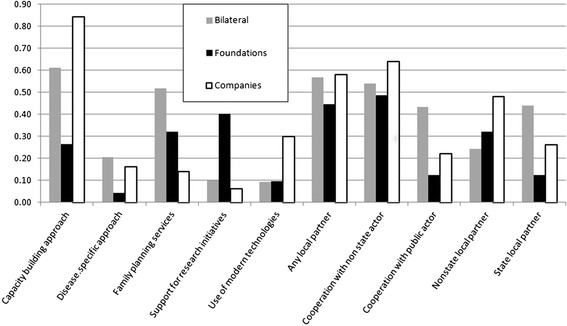
**Substantive and cooperative dimensions of donor projects (in % per donor type).**

The following section provides an overview of the different maternal health projects by donor groups using the different categories of analysis. As can be seen from Figure 1 and Table 2, donor groups differ substantially in most aspects of maternal health programs. Regarding some aspects, however, within-group variation is often high indicating small between-group differences.

**Table 2:** **Estimates of donor effects on the substantial dimension of projects**

	**Family planning services**	**Disease specific approach**	**Capacity building approach**	**Use of ICT**	**Support of research initiatives**
Random effects					
Between donors (within donors)	1.07 (1.03)	0.71 (0.84)	1.02 (1.01)	1.44 (1.20)	1.24 (1.11)
Fixed effects					
(Intercept)	−0.14 (0.50)	−1.83*** (0.47)	0.70 (0.50)	−3.68*** (0.80)	−2.67*** (0.62)
Corporations	−1.31^#^ (0.77)	0.09 (0.75)	1.42^#^ (0.85)	2.71** (1.05)	−0.30 (1.06)
Foundations	−2.36** (0.90)	−1.50^#^ v	−1.85* (0.74)	1.31 (1.05)	2.66** (0.85)
AIC	310.2	207	288.7	184.4	205.6

*Note:* Values are GLMER coefficients; standard errors are in parentheses.

***p < 0.001, **p < 0.01, *p < 0.05, ^#^p < 0.1.

Figure 1 and Table 2 highlight the substantial dimensions of maternal health projects. The first aspect involves family planning services. As it turns out, 42% of all donors include family planning services in their projects, with bilateral donors comprising the highest amount (52%). In comparison, foundations and corporations included significantly less family planning services in their project portfolios (see Table 2). 32% of foundations’ projects and only 14 percent of projects by corporations included such services (see Figure 1).

Twenty-one percent of bilateral donors’ projects are disease-specific, in comparison to 16 percent of corporate projects and four percent of foundation projects. Only the difference between bilateral donors and foundations is statistically significant. On the individual donor level, the United States stands out, as almost half of their many projects (19 of 41) are disease-specific. A more detailed analysis shows that those are mostly interventions to address malaria in pregnancy, nutrition problems during pregnancy, maternal anemia and postpartum hemorrhage. USAID’s Maternal and Child Health Integrated Program (MCHIP) uses “evidence-based interventions” to improve maternal health in four main areas that are mostly disease-specific: prevention of postpartum hemorrhage; prevention and treatment of preeclampsia/eclampsia; prevention of maternal anemia, and expanding access to and improving the capacity of skilled birth attendants. They also aim to promote the integration of FP, malaria and HIV/AIDS activities within maternal health programs [26]. Excluding USAID significantly reduces the percentage of disease-specific projects in the group of bilateral donors, resulting in a figure only half as high.

Concerning capacity-building, bilateral donors (61%) and especially corporations (84%) have a stronger focus on this area (Figure 1). In contrast, only 26% of foundation projects involve capacity-building. In general, it is noteworthy that over half of all donors (56%) include capacity-building components. Looking at private donors, exactly 50 percent of their projects include capacity-building. Within the group of bilateral donors, the UK stands out, with only 20 percent of their projects including capacity-building. All other donors have percentage shares of 60 percent or more. As for foundations, half of the projects of the Hewlett and Ford Foundation have capacity-building components, but the remaining foundations have little mention of capacity-building. All of the corporate donors, on the other hand, have these components in three quarters or more of their projects.

Few maternal health projects have a focus on implementing ICT. This is particularly true for bilateral donors, from which only the US included ICT components in their projects (about 33%). One third of all corporate donors also work in this area compared with only ten percent of foundations. Most of USAID’s ICT interventions in maternal health focus on using mobile technology to deliver behavioral change messages to mothers or mentoring to maternal health workers, through public-private partnerships such as MAMA (Mobile Alliance for Maternal Action) or the mHealth Alliance (mPowering Frontline Health Workers). The Gates Foundation included ICT in only two of their 13 projects. Except for the Abbott Fund and Johnson & Johnson, all corporate donors are strongly involved in ICT projects, especially the Novartis Foundation, through interventions such as e-learning tools or ICT referral systems in rural areas. This is underlined by the fact that the use of new technologies such as e-learning or mHealth is one of the foundations’ five approaches, adopted to deploy a business mindset and outcome-based thinking [27].

Eighteen percent of all donors include a research component in their projects. Foundations incorporate a research aspect in 40 percent of their projects. The percentage share for bilateral donors is ten, and for corporate donors six. As for individual bilateral donors, Australia (25%) and the US (17%) conduct some research projects. In contrast, all foundations are involved in research projects. The coefficient for foundations is highly significant. All of the Wellcome Trust’s projects involve a research component, since health research is the main focus of the organization. Furthermore, the Hewlett and the Gates Foundation are also heavily engaged in research. Even though the number of research projects by foundations is already very high, it could have been even higher if global research alliances, networks or development partnerships without concrete projects would have been included. The Gates Foundation’s commitment to research, in particular, focuses on the development of new drugs, vaccines and diagnostics, which is generally not included in this analysis as this does not occur at a project level [28].

Figure 1 and Table 3 highlight the partnership dimension of maternal health projects. Partnerships with local and international organizations are prevalent in most projects. Looking at all donors together, 53 percent of all projects involve a local partner. This is a relatively low number, considering the 2005 Paris Declaration on Aid Effectiveness which aims to achieve more national ownership. All donors work almost equally together with state (23%) and non-state partners (21%), although with a much smaller combination of both (9%). Fifty-seven percent of bilateral donors and only 44 percent of foundations work together with local partners, in comparison to 56 percent of corporate donors. 49 percent of private donor projects have a local partner, which is less than with public actor projects. As for bilateral donors, the most common local partners are state institutions (33%), followed by NSAs (13%) and a combination of state and non-state partners (11%). However, these differences are statistically not significant (see Table 3). The random effects indicate that the between donor variability of having a local partner is rather low and superimposed by a higher within donor variability. That means that most donors do not show a clear profile themselves.

**Table 3:** **Estimates of donor effects on the cooperation dimension of projects**

	**Any local partner**	**Local partner non-state**	**Local partner state**	**Cooperation non-local NGO**	**Cooperation non-local state**
Random effects					
Between donors (within donors)	0.86 (0.93)	0.47 (0.68)	2.85 (1.69)	1.19 (1.09)	1.56 (1.25)
Fixed effects					
(Intercept)	0.45 (0.46)	−1.38*** (0.38)	−0.18 (0.78)	0.15 (0.54)	−0.37 (0.59)
Corporations	0.15 (0.73)	1.39* (0.59)	−0.95 (1.20)	0.51 (0.82)	−0.90 (0.93)
Foundations	−0.76 (0.68)	0.30 (0.57)	−2.12^#^ (1.18)	−0.31 (0.77)	−1.80^#^ (0.92)
AIC	347.8	315.8	263.2	324.2	292.9

*Note:* Values are GLMER coefficients; standard errors are in parentheses.

***p < 0.001, *p < 0.05, ^#^p < 0.1.

The most common local partners of foundations are non-state institutions (32%), with state actors only being involved in 13 percent of the projects. Corporations work with almost the same amount of states (10%) and NSAs (30%) as foundations, but work more with a combination of both (16%) and therefore have a higher share of local partners.

Regarding local partners, corporations work significantly more with non-state organizations than bilateral donors (see Table 3). Especially the Novartis and Sanofi Espoir Foundations have many partnerships with non-state organizations. Similarly, foundations work significantly less with public partners. Two foundations in particular have noteworthy partnership strategies (see Table 4): the Gates Foundation involves local partners in only 8 percent of their projects, by far the lowest number of total local partner involvement of all 15 donors. The Ford Foundation, in contrast, gives 73 percent of their overall grant-making in this area to local institutions, most of them also non-state. This confirms the findings of a study by Chervalier and Zimet [29], which compares twelve foundations and their grant recipients. According to them, the Ford Foundation favors direct partnerships with institutions in the global South, in comparison to the Gates, Hewlett and Packard Foundations. Differences between foundations and bilateral donors with regard to involvement with local non-state organizations as compared to corporations and bilateral donor’s involvement with local public organizations are statistically not significant.

**Table 4:** **The cooperation dimension of projects (in % for each organization)**

	**Any local partner**	**Local partner non-state**	**Local partner state**	**Cooperation non-local NGO**	**Cooperation non-local state**
	**%**	**%**	**%**	**%**	**%**
Australia	60	10	55	25	70
Canada	32	16	16	60	40
Germany	93	13	80	27	13
United States	76	23	18	27	45
United Kingdom	36	42	68	100	42
Ford Foundation	73	60	13	33	0
Gates Foundation	8	8	0	69	31
Hewlett Foundation	42	8	33	58	0
Packard Foundation	46	46	0	50	4
Wellcome Trust	50	0	50	17	67
Abbott Fund	50	50	0	100	0
Johnson Johnson	36	36	0	64	55
Merck	50	42	8	67	4
Novartis	75	50	75	50	75
Sanofi	100	78	89	56	11

All donors work with non-local, non-state partners. Corporations do so for most projects (64%), followed by bilateral donors (54%) and foundations (49%). Since within-donor group variance is quite large, these results are statistically not significant. The random effects indicate that the between donor variability of having a local partner and the within donor variability are almost equally high. As a result some donors as well as donor types have no clearly defined profile. Regarding bilateral donors, the United States conducts all its projects with a non-local, non-state partner. All USAID’s Maternal and Child Health Integrated Program projects are conducted by a core set of partners that come from a pool of eight NGOs or corporations. More concretely, all projects involved the partner Jhpiego, and very often also PATH, Save the Children and John Snow Inc. USAID further collaborates with many additional partners, such as foundations and corporations in public-private partnerships. As for Canada, non-local, non-state partners were mostly Canadian NGOs. These two bilateral donors confirm the assumption that many bilateral donors have long-standing relationships with their home-country development actors as the main intermediaries for funds to support development. However, not all bilateral donors work in the same way; Australia, Germany and the UK only work with home-country NSAs in one third of their projects. Hence, the high number of bilateral projects with non-local NSAs is largely driven by the US and Canada. Foundations worked with the least amount of non-local NSAs, although they still work with many. Their usual types of non-state partners are akin: mostly international NGOs, a few private research institutions and very few corporations. Many of these partners are US-based, as are four of the five foundations.

Thirty-one percent of all donors’ projects involve a non-local public partner. Bilateral donors lead the ranking (43%), followed by corporations (22%). Foundations only have very limited cooperation with non-local public donors (13%). The difference between foundations and bilateral donors is statistically significant. Bilateral donors all work with non-local public actors; some less (Germany, 13%) and some more (Australia, 70%). In the case of Australia, those public donors are other bilateral agencies or multilateral donors. As for Canada, the UK and the US, public partners are mostly multilateral organizations (e.g. UNICEF, WHO, World Bank). Regarding foundations, the Wellcome Trust works with non-local public actors in two thirds of their projects. The Gates Foundation does so in one third, and the remaining three foundations work with (almost) no non-local public partner. By excluding the Wellcome Trust, only eight percent of foundations’ projects have a non-local public partner. Finally, concerning corporate donors, the Novartis Foundation and Johnson & Johnson work extensively together with non-local public actors (75% and 55%, respectively). However, Novartis only conducted four projects in total. Multilateral organizations could be a preferred partner for the same reasons, as they have established field presence in many countries. This category can be compared to the number of local public partners. By looking at all donors together, more work with non-local (31%) than with local public partners (23%). This may be due to a reliance on home-country public research institutions, other donor agencies and multilateral organizations – all important actors in the patchwork of international donors. The largest difference is in the group of corporations (22% non-local vs. 10% local partners) followed by bilateral donors (43% non-local vs. 33% local partners). The foundations’ work with non-local and local public partners is equally distributed (13%). At an individual level, exceptions are in Germany (80% local and only 13% non-local public partners), with the Gates Foundation (31% non-local and no local public partners) and with the Abbott Fund (no public partner at all).

Figure 1 shows that the majority of all projects rather included non-state partners than public actors (69% compared to 51%). Especially the private actors did so, with foundations showing the largest difference.

## Discussion

In the results section, we identified typical profiles of donor project characteristics. Bilateral donors can be characterized as focusing on family planning services, specific diseases and capacity-building while, in comparison, disregarding research and ICT. Bilateral donors cooperate with local public authorities and with governments and NGOs from other developed countries. In contrast, corporations focus their donor activities on specific diseases, capacity-building and ICT while disregarding family planning services and research. Corporations work together with local and in particular with non-local non-state actors. Foundations can be characterized as focusing on family planning services and research, while disregarding specific diseases, capacity-building and ICT. Foundations cooperate less than other donors; but when they do, they cooperate in particular with non-state actors, local as well as non-local.

These profiles partly match donor funding patterns. Regarding bilateral donors, development funding has changed in the last decade. In particular, the development discourse of government aid agencies and international organizations has altered preferences of bilateral donors [30]. Historically, governments focused their assistance on specific countries and regions on grounds of economic and political interests, historical ties or geographical proximity. Since the end of the cold war, the preferences have shifted towards perceived needs of developing countries. The intention was to have a lasting impact on the root causes of suffering, rather than alleviate suffering in the short term. As a result, a capacity-building approach became the strategy of choice, rather than handing out, for example, food aid [30]. Additionally, the sector-wide approach (SWAP) emerged in the 1990s, a specific model of cooperation in which donors agree to pool resources within some specific sector, supporting a single sector policy and expenditure program under government leadership [31]. Recent developments, however, indicate that bilateral donor governments are increasingly being challenged by taxpayers in their countries to become more accountable and transparent in their spending practices. At the bilateral level, donors are beginning to restrict health aid flows, putting renewed emphasis on impact, co-financing and value for money [4]. As a result, vertical (disease-specific) programs have reemerged and donors increasingly support these programs because these programs are easier to monitor and evaluate, and their results are available more quickly [32]. Furthermore, McCoy and Kinyua find no relationship between total health expenditure and government expenditures on health of a recipient country and the amount of funding granted by bilateral donors [5]. According to a WHO Bulletin paper, half of the additional funding between 2000 and 2009 targeted only two diseases: HIV and malaria [33,34]. Maternal health projects mirror these developments. Bilateral donors focus on capacity-building and, at the same time, on disease-specific projects. This combination ensures long-term effects of bilateral aid and makes effects measurable as is the case with disease-specific projects [35].

Regarding family planning services, the historical rationale for public involvement in family planning (FP) provision is based on the negative externalities associated with high rates of population growth. For example, the United States has a strong history of supporting FP as part of its foreign assistance programs [19]. However, this support has been fraught with challenges and there has been an enduring gap in US policy approaches on this issue [19]. According to Ruth Levine, USAID’s FP program has suffered more than any other program from being a “political football” [36]. This has, among other factors, been caused by the perceived linkage of FP to the highly charged issue of abortion. However, as it turns out, we did not detect a “political football” effect for family planning services. Quite the contrary, even the US projects involve a family planning component in most instances which includes family planning counseling and training with local health care workers. It seems that US projects avoid taking inroads into the social and political minefield on the issue of abortion.

Regarding cooperation with local and foreign organizations, public donors often have well-established relationships with particular developing countries that provide a basis for dialogue and cooperation, written down in official country agreements [37]. This form of cooperation is further shaped by the principles of current aid strategies based on the 2005 Paris Declaration. Two of the five principles in the Declaration are particularly important to mention here: national ownership and alignment [38]. For many bilateral donors, civil society is seen to play an important role in helping build country ownership of aid policies, as they have come to realize that national ownership should mean more than state ownership. Two developments support this trend. First, many donors are looking for more in-country contextual analysis and many mention the importance of conducting mapping exercises, in order to better understand the social and political landscape of recipient countries and make more informed partner choices. Second, due to this emphasis on southern civil society and the desire to fulfill the Paris Declaration principles, a rising interest in establishing in-country multi-donor funding mechanisms can be observed. These ‘pooled funds’ go to locally contracted and granted projects in the south, often to fund civil society organizations [38].

Philanthropic donors are extremely diverse but share some common characteristics. First, foundations are “perhaps among the most unaccountable organizations in democratic societies”, having no voters, shareholders or customers [1]. In comparison to NGOs and corporations, they are not dependent on donations and do not have to compete on a market [30]. This is the reason for their signature characteristic: the capacity to be innovative and creative, take risks, act as an alternative to the state and provide long-term, unrestricted support for those ‘beyond’ the market and the state [1,3,6]. As our results indicate, research-based activities are indeed a primary focus of philanthropic donors, which to our surprise do not involve much ICT.

Corporate donors have shareholders and customers and are therefore not as independent as foundations. Key components that influence corporate giving priorities are enhancing corporate image, increasing brand recognition, introducing new products into the market, giving back to communities where they have a presence and, also, identifying a niche [13]. Corporations are largely seen to be concerned with issues that directly benefit the corporation’s stakeholders or promote the corporation’s business objectives [39,40]. Generally, it has been argued that as private donors do not need to consider priorities such as foreign policy, they can hence select projects on the basis of need and focus on problem-solving [6]. As with business, private donors also like to know the impact, including social returns, of their investments in development. Hence shareholders demand development results [41]. These two issues, the problem-oriented behavior and the will to be able to show results, lead to a trend of keeping overheads low and identifying key indicators of performance and impact. It is the corporations’ goal that their funding and efforts have the greatest impact possible [6]. This ‘business-like approach’ encourages the transfer of successful strategies, management techniques and principles from the for-profit sector to international development. They emphasize solving problems, taking risks, fostering innovation and measuring success [6]. Private donors are therefore increasingly focused on finding technical solutions to clearly identified problems, also leading to vertically delivered, disease-specific programs [41]. They often return to projects merely providing equipment or technology, e.g. vaccinations, new water systems or bed nets. Maternal health projects confirm these studies, in that their projects are disease-specific and have a focus on technical solutions such as ICT. However, the findings of Marten and Witte, that corporations show a preference for vertically-organized programs due to dysfunctional local structures that project implementers sometimes face [6], cannot be confirmed here. Corporations include capacity-building in 4 out of 5 maternal health projects.

Foundations and corporations usually work outside of state structures and, by and large, their programs are not connected to national development strategies. Therefore, if they have local partners, these are mostly non-state partners [6,40]. However, private donors often struggle with the complexities of finding capable and suitable partners on the ground with whom to work [3]. As a result, they show a preference for non-local NGOs. The reasons for this can be found in strong relationships to home-country non-state development actors such as grant recipients and in problems with finding suitable partners in the project country. This raises questions of local ownership and alignment with national policies. Skeptics point out that the marginal involvement of recipient country governments raise questions of sustainability. In terms of process, it can be argued that in some cases, bypassing the government is an unsustainable approach [2]. Working with governments and thus, strengthening the health system, might be the most efficient in the long term. However, this is difficult in the short term, and therefore, bypassing government and going straight to communities can potentially result in more immediate impact [2]. Private donors prefer quick results and measuring success, which can explain the preferred partner choice. However, it needs to be stated that there is less but, of course, still some cooperation with local and public actors.

There may be limitations to our findings. First, our study focuses on the largest donors regarding maternal health programs. We argue that large donors exert influence on the direction and the priorities of aid. However, this does not mean that smaller donors have similar priorities in terms of e.g. cooperation partners or ICT utilization. Second, our sample has a strong bias towards the US and the Anglosphere. As a result, variation between donors might be largely driven by cultural or political factors peculiar to the Anglo-Saxon and US world. It is plausible to assume that donors from other countries such as France or China might have different priorities. Further research that includes a more diverse set of donors is necessary to tackle questions of cultural and political factors underlying aid funding as well as project objectives. Third, underlying our analysis is a donor perspective which focuses on donor characteristics as an explanatory variable for project priorities. However, projects might also be recipient driven in that certain recipients and problems require certain project components. Fourth, another limitation of our study is that our outcome measures are self-reported and therefore be subject to biases such as a social desirability and reporting style. Some donors provided extensive information for each project while others are more reluctant to make information available. This problem could be solved by conducting interviews with representatives of the donor organizations in order to help establish a greater degree of accuracy on this matter.

## Conclusions

Our analysis reveals that there are notable differences between donor groups regarding the implementation of maternal health projects. Our theoretical expectations that were derived from numerous studies focusing on funding patterns were only partly confirmed. This suggests that project implementation follows somewhat different rules than mere funding. Maternal health is a central point in funding and project development of corporations and foundations, but not of bilateral donors. Therefore, it is premature to generalize beyond the issue of maternal health. Theoretically, it would be interesting to know if funding and project implementation vary systematically over all issues and which factors account for the variation. Furthermore, future studies should provide donor classifications on the basis of funding and projects to give a more accurate picture of development and health aid. Our analysis lends itself for a characterization of implementation styles of different donors. These implementation styles give rise to some policy recommendations:

Since donor groups specialize in different contexts, NGOs and governments working on development and health aid may target donors groups that have specialized in certain issues.

There is a need for information-sharing between donor groups in order to combine the project portfolio of each donor group. This also enhances the possibility space of projects. These can be recombined to find the most appropriate solution to fight a given problem in health and development policy.

The establishment of a global database with uniform spending data of foundations and other private actors would significantly help us to understand the increasingly important role of private donors as direct operators, grant-givers or as members of a partnership.

Private aid is offering new business models and transformative technologies which can complement the approaches of traditional donors. There is reason to believe that both these strategies will provide good development assistance, but only if coordination prevails. This coordination needs to be based on the above-mentioned information-sharing. When one actor is working in a particular niche, he has specific knowledge and knows about best practices and lessons learned. The global aid architecture should endorse these specializations and coordinate between certain actors. This has partially already been done, for example, in disease-specific networks that try to bring together all relevant actors working in a specific field.

## Endnote

^a^This limitation of the sample selection procedure was mentioned by one of the reviewers, which we gratefully acknowledge.

## Abbreviations

ICT: Information and communication technology
GLMER: Generalized linear mixed-effects models
NGO: Non-governmental organization
WHO: World Health Organization
OECD/DAC: Organisation for Economic Co-operation and Development's Development Assistance Committee
IDS: International Development Statistics
ODA: Official Development Assistance
FP: Family planning services
NSA: Non-local non-state actor
